# CT diagnosis and destiny of acute aortic intramural hematoma

**DOI:** 10.3389/fradi.2025.1552644

**Published:** 2025-03-11

**Authors:** Giacomo Sica, Gaetano Rea, Roberta Lieto, Mariano Scaglione, Ahmad Abu-Omar, Giorgio Bocchini, Federica Romano, Salvatore Masala, Stefania Tamburrini, Salvatore Guarino, Candida Massimo, Tullio Valente

**Affiliations:** ^1^ Department of Radiology, Azienda dei Colli, Monaldi Hospital, Naples, Italy; ^2^ Department of Medicine, Surgery and Pharmacy, University of Sassari, Sassari, Italy; ^3^ Department of Emergency Radiology, University of British Columbia, Vancouver General Hospital, Vancouver, BC, Canada; ^4^ Department of Radiology, Ospedale del Mare-ASL NA1 Centro, Napoli, Italy

**Keywords:** aortic intramural hematoma, computed tomography angiography (CTA), acute aortic syndrome (AAS), penetrating atherosclerotic ulcer (PAU), aortic dissection (AD), diagnosis

## Abstract

Acute aortic intramural hematoma (IMH) is a relatively uncommon but potentially life-threatening aortic disease that can occur primarily in hypertensive and atherosclerotic patients. The course of IMH varies widely, with the condition either regressing, remaining stable, or progressing until it leads to outward rupture or intimal layer disruption, eventually resulting in overt aortic dissection. Therefore, poor prognostic computed tomography (CT) features must be promptly recognized and reported by the radiologist. In emergency departments, readily accessible non-invasive CT angiography is crucial for achieving a rapid and accurate diagnosis essential for appropriate management. For Type A and B aortic dissection, surgery is typically recommended in Western countries for patients with Stanford Type A IMH and those experiencing irrepressible pain. For Stanford Type B IMH patients without complications or incessant pain, medical treatment is suggested but with imaging follow-up. In complicated Stanford Type B situations, thoracic endovascular aortic repair (TEVAR) is currently indicated. This review aims to present pathophysiology, CT diagnosis, and IMH fate and provide the reader CT image-based review of the CT diagnostic criteria, complications, and associated critical prognostic findings of this rather rare aortic disease.

## Introduction

Acute aortic syndrome (AAS) encompasses overlapping, interchangeable, and life-threatening diseases, such as acute intramural hematoma (IMH). IMH is characterized by a small hematoma (≥5 mm) within the aortic wall's media, without a visible intimal tear or intimomedial flap (1–3). IMH constitutes 5%–27% of AAS cases and can develop spontaneously as an isolated event (90%) or be associated with penetrating atherosclerotic ulcer (PAU, 5%) or result from post-traumatic or iatrogenic aortic injury (2, 4–6). Asian cohorts have shown a greater incidence of IMH compared to the International Registry of Acute Aortic Dissection series, with rates of 28.3% vs. 3.6%, respectively. Additionally, these groups have higher rates of early medical treatment and lower overall mortality (6–12). Sudden tearing chest or back pain and chronic hypertension are common clinical presentations associated with AAS entities. However, in IMH, these symptoms and typical findings may be absent or subtle, depending on the extent of the anatomic lesion and the involvement of adjacent vascular structures.

A clinically high index of suspicion is essential to identify the disease and request appropriate imaging studies for prompt diagnosis, which can provide crucial life-saving indications for timely treatment. Indeed, IMH continues to be frequently misdiagnosed today, with mortality rates ranging from 10% to 50% and progression to overt aortic dissection (AD) in over 40% of patients (13). The most common location of IMH is the descending thoracic aorta (Type B IMH, 60%–70%) followed by the ascending aorta and aortic arch (Type A IMH, 30% and 10%, respectively) (2, 13). Rapid and non-invasive computed tomography (CT) angiography allows for correct diagnosis in most cases, with sensitivity and negative predictive value approaching 99% (2, 14–16). The evolution of IMH involves a hyperacute phase (<24 h), an acute phase (2 weeks from pain onset), a subacute phase (2–6 weeks), or a chronic phase (>6 weeks) (10, 15, 17). Usually, treatment and diagnosis for IMH are similar to those recommended for AD based on the segment of the aorta involved (Stanford classification) (2, 7, 17, 18). However, given the highly variable and often unpredictable natural history of IMH, the prognosis and management largely depend on several key morphologic features that can be identified through CT imaging.

## Etiologic controversies and IMH pathogenesis

The etiology of IMH remains controversial, with no single recognized cause identified to date. In 1920, Krukemberg was the first to propose rupture of the vasa vasorum as the initial event of the AD process (19). Later, Gore proposed that underlying degeneration of the media could be a predisposing factor for vasa vasorum hemorrhage and IMH (20). Historically, IMH was believed to emerge from rupture or rhexis of the vasa vasorum within the aortic media, linked to the atherosclerotic process and systemic hypertension, where increased stress on the aortic wall contributes to medial hemorrhage without intimal disruption (20–22).

However, the development of increasingly high-performance CT scanners and the significant increase in their spatial resolution, associated with ever-increasing clinical experience, have allowed us to focus our attention on the possible progressive focal rupture of the intima, called ulcer-like projection (ULP) found by imaging tools in 20%–50% of IMH and considered the “primum movens” that leads to the formation of IMH over time (6, 8, 22–28).

Conversely, other explanations suggest that ULP may develop over time from IMH, as intimal disruption can occur progressively in the acute or subacute phase, leading to the appearance of ULP after focal intimal disruption by the IMH. ULP corresponds to a focal contrast enhancement in the thickness of the IMH communicating with the aortic lumen, in the absence of signs of atherosclerosis or calcification (8, 29).

Currently, the belief that IMH is an AD without intimal rupture is increasingly strong and that therefore this can be considered a variant or precursor of AD. Moreover, some patients may present with both lesions in different segments of the aorta simultaneously. Essentially, in IMH, a thrombus separates and fills the layers of the aortic wall (probably because IMH has an entry tear without re-entry) rather than free-flowing blood as seen in typical AD. Therefore, IMH cases are essentially cases of acute AD or AD with an acutely closed and thrombosed false lumen, representing a non-communicating type of AD (24–26). Several studies also report a coexisting penetrating atherosclerotic ulcer (PAU) in 50%–90% of cases in acute IMH (28, 30–34).

**Figure 1:**
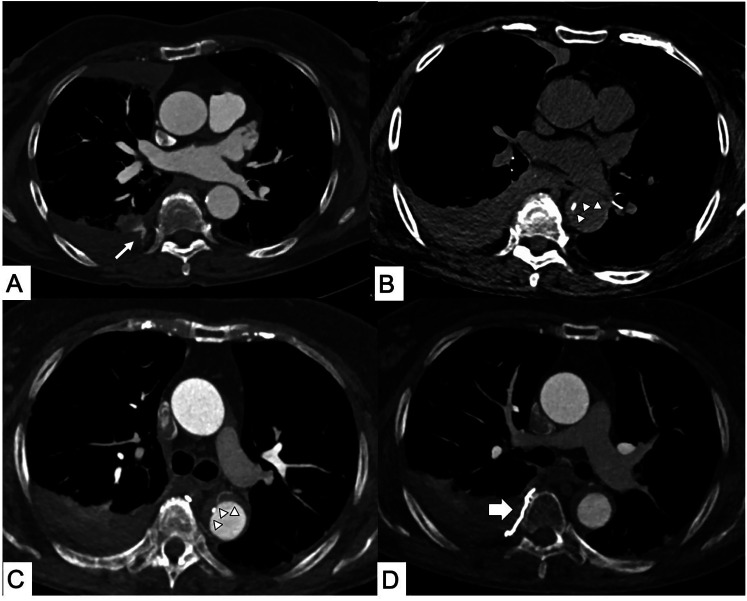
Iatrogenic intramural hematoma (IMH). Computed tomography (CT) axial scans. **(A)** Right intercostal active bleeding after lung biopsy (thin arrow). **(B)** Axial CT performed for chest pain 1 day after the intercostal artery transcatheter embolization procedure shows the appearance of hyperattenuated aortic wall thickening from IMH with **(C)** displacement of intimal calcification and bronchial artery in the contest of the Type B IMH. **(D)** No more active bleeding in the site of embolization (thick arrow).

On histological analysis, IMH is predominantly intramedial, although in certain segments of the aorta, it may be subadventitial (between the media and the adventitia), indicating a contained rupture of the aortic wall. Less commonly, IMH can result from a PAU involving the internal elastic lamina (21, 22, 34) or from blunt or iatrogenic traumatic injuries to the aortic wall, such as during percutaneous catheterization, angioplasty, placement of a balloon pump, or left side catheter ablation (Figure 1).

## CT angiography technique

In the recent Guidelines for Diagnosing and Treating Acute and Chronic Syndromes of the Aortic Organ (7), authors reiterated minimum technical standards capable of guaranteeing diagnostic reliability: an isotropic resolution of 1 mm or less, ECG gating (for the study of the aortic root and ascending aorta), and fast acquisition techniques to reduce pulsation artifacts increasing measurement accuracy for any surgical planning.

The latest dual-energy CT scanners, such as photon-counting CT scanners, enable image acquisition with an isotropic spatial resolution of 0.2 mm, a high temporal resolution of 66 ms, and a coverage of 2 m in <3 s. These advancements promise to significantly enhance the visibility of the arterial wall in CT images and improve diagnostic quality, while also reducing the volume of contrast agent needed through low-keV virtual monoenergetic reconstructions, and minimizing the number of acquisitions and radiation exposure by using virtual non-contrast imaging derived from a contrast-enhanced acquisition (35–42).

**Figure 2:**
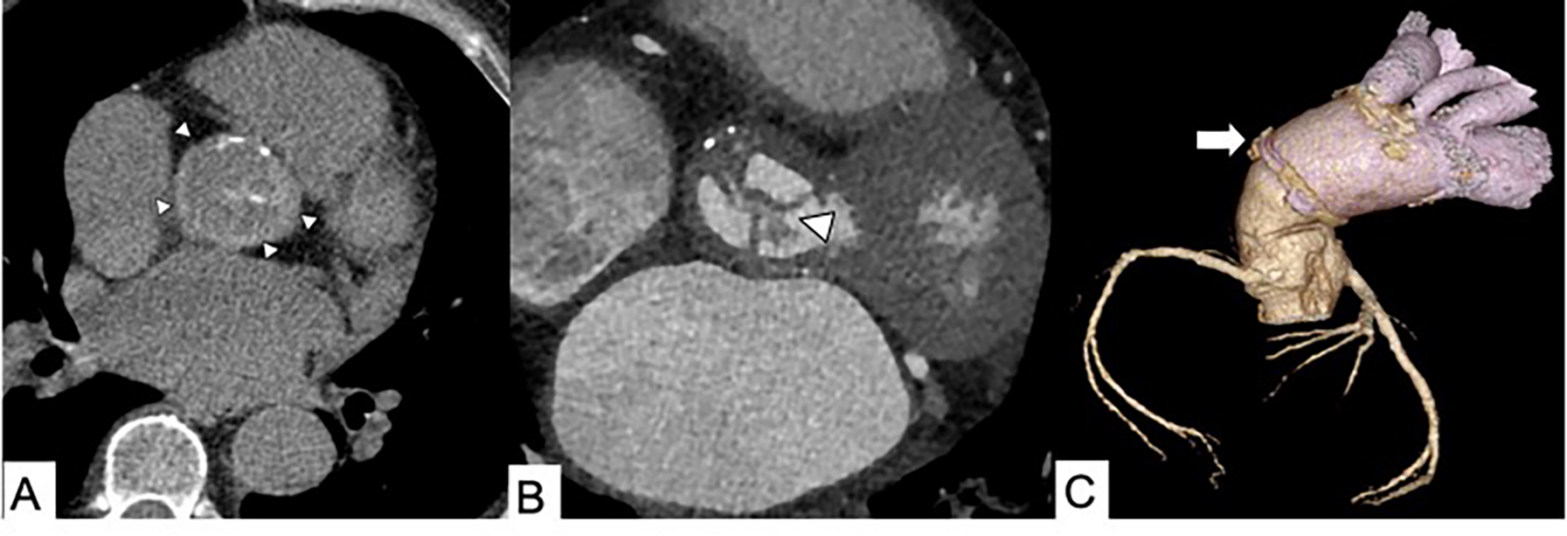
Infected endocarditis in a 65-year-old male patient with aortic bioprosthetic valved conduit. **(A)** A non-enhanced axial CT scan shows a hyperattenuating aortic prosthetic wall (arrowheads). **(B)** Thickening of prosthetic valvular leaflets with endocarditis vegetation (arrowhead) and **(C)** a 3D volume rendering reconstruction showing distal graft aortic anastomosis (arrow).

In cases of suspected AAS, our institution utilizes a CT protocol that begins with a helical high-pitch non-contrast scan. This scan is crucial for distinguishing aortic wall thickening in IMH from that due to other causes, and it helps identify hemopericardium or newly thrombosed blood, as well as surgical materials in patients with a history of cardiac surgery (Figure 2). Following this, a high-pitch prospective ECG-gated arterial-phase scan is performed to obtain motion-free images not only of the aortic root and thoracic aorta but also of the coronary arteries, thus allowing the evaluation of the health status of the coronary arteries without subjecting eventually the patient to invasive coronary angiography (7, 35). A subsequent portal venous phase can help assess malperfusion of the abdominal organs. A high iodine concentration of contrast medium (CM) (370–400 mgI/ml) can improve contrast enhancement and image quality to allow the identification of even the tiniest intimal disruptions possibly associated with IMH (43–45).

Getting high intravascular attenuation is essential for having high-quality diagnostic images. Several factors influence the attenuation of vascular structures, including patient size and circulation time, but above all, a good contrast medium injection protocol is influenced by the concentration and volume of the contrast medium as well as the injection rate. Vascular enhancement is in fact directly dependent on the iodine concentration of the contrast medium and the injection rate, represented as the iodine delivery rate (IDR). IDR represents the iodine load administrated per unit of time and can be evaluated by applying the following formula IDR = (*I*/1,000) × FR where *I* is the contrast medium (CM) iodine concentration (mgI/ml) and FR is the CM injection flow rate (ml/s). It is possible to increase IDR either using high-concentration CM or increasing the FR.

The total amount of contrast depends on the protocol adopted. In the case of only the angiographic phase, we calculate it with the formula Vcm = (Scan duration + delay) × FR. In the case of suspected organ malperfusion, to be also studied with a portal phase, the total amount of contrast media needs to be calculated based on the body weight of patients and the concentration of contrast media. At our institution, we follow the recommendations of our scientific society, the Italian Society of Medical Radiology, maintaining an IDR between 1.6 and 2.2 grI/s. Furthermore, intravascular attenuation can be increased by using tube potentials of 100 kVp, lowering it up to 80 kVp in small patients, combined with an iterative reconstruction algorithm, thereby reducing the need for higher concentrations and volumes of iodinated contrast medium (46–48).

## Ultrasound notes

CT has become the preferred modality for evaluating most patients with suspected AAS due to its wide availability, its quickness to perform, and its panoramic view capable of evaluating the entire aorta and any signs of malperfusion, pericardial or pleural effusion, and periaortic or mediastinal hematoma. Nevertheless, at our institution, AAS initial diagnosis is always constituted by an integration between transesophageal echocardiography (TEE) if transthoracic echocardiography (TTE) is suspicious and CT angiography. In general, echocardiography is an advantageous method because it can be performed at the patient's bedside in whatever department he is located and even if he is in an unstable hemodynamic situation. While TTE should be used routinely in clinical practice, TEE requires greater knowledge and experience. Echocardiographic IMH features include an intramural echo-free space, a crescentic or circular wall thickness of >7 mm, and an aortic lumen or intimal calcification displacement caused by media intramural hematoma that is useful for the differential diagnosis; the inner margin of IMH is smooth and aortic thickening occurs beneath the bright echo-dense intima. However, differentiating an IMH from an AD with a thrombosed false lumen can be very challenging. Echocardiographic artifacts yield a significant number of false positive results, particularly in the ascending aorta (49, 50).

## CT diagnosis

Normal aortic wall thickness is <3 mm. Evaluation of IMH thickness is usually based on axial measurements or perpendicular to the longitudinal axis of the aorta, measured between the outer walls of the vessel.

**Figure 3:**
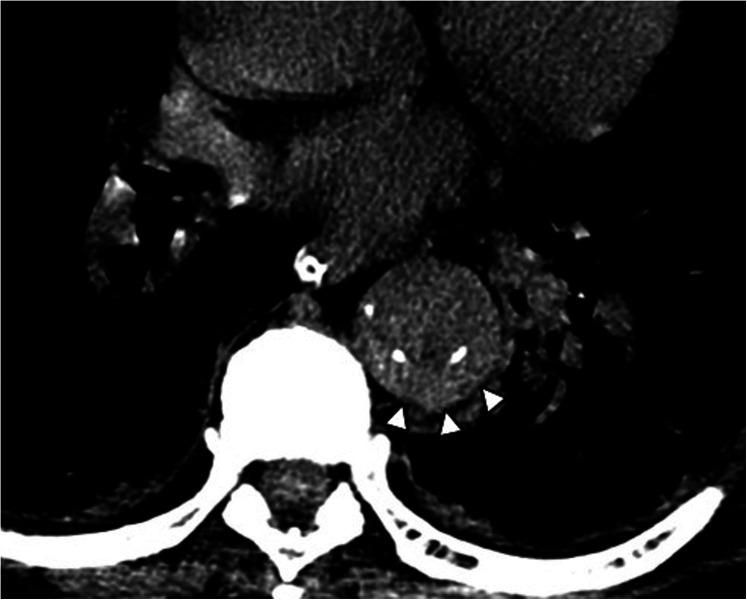
Non-contrast CT axial image in a 73-year-old female patient shows the diagnostic hallmark of IMH: displacement of intimal calcifications and hyperattenuating crescentic thickening of the aortic wall (arrowheads).

**Figure 4:**
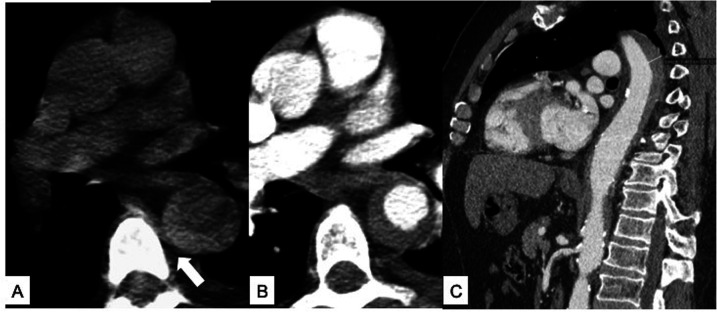
CT scan in a 62-year-old male patient with acute chest pain and Type B IMH. **(A)** Axial non-enhanced CT image shows hyperattenuating crescentic thickening of the aortic wall (arrow). **(B)** Axial contrast-enhanced CT scan shows a reduced diameter of the aortic lumen and the slick interface between the lumen and wall. **(C)** Contrast-enhanced sagittal multiplanar reformation CT image shows a reduced diameter of the aortic lumen and its constant circumferential relationship with the wall.

Unenhanced CT images play a crucial role in establishing the diagnostic hallmark, characterized by circular or crescent-shaped, non-spiral, hyperattenuating (60 ± 15 HU) wall thickening of ≥5 mm (7, 45, 51). This may include centripetal displacement of intimal calcification (Figure 3) and a certain degree of narrowing of the aortic lumen, although the luminal-wall interface remains smooth. By definition, both an intimal flap and double channel intraluminal flow are absent in IMH. Post-contrast images alone can result in false-negative interpretations due to masking from the high-attenuation contrast in the adjacent vascular lumen. Additionally, the hematoma appears similar on contrast CT, with no enhancement of the thickened wall. IMH can be distinguished from AD not only by the absence of an intimal flap and tear but also by its relatively constant circumferential relationship with the wall, as opposed to the spiral configuration commonly observed in AD (Figure 4).

The longitudinal extent of IMH can vary greatly, ranging from very short (∼1 cm) to extending the entire aorta. In the presence of Type A IMH, the space between the aortic lumen and the right atrial appendage, typically closely contiguous structures, increases due to the presence of aortic wall thickening (52, 53). To monitor the possible progression of the disease, it is essential to report at the time of diagnosis the minimum and maximum axial diameters of the aorta involved in the hematoma (54, 55).

MRI techniques allow for the assessment of the age of the hematoma by detecting methemoglobin formation within the IMH, leading to increased signal intensity on T1-weighted images in subacute IMH (56, 57).

The widefield view provided by axial imaging, aided by multiplanar reformations (MPR), is crucial for accurately defining the extension of the hematoma and periaortic bleeding. Common findings in IMH also include extravasation of fluid, mediastinal hemorrhage, and pleuropericardial effusion (58).

## Natural history, IMH fate, and evolutive patterns

Acute Type A IMH involving the ascending aorta can be a life-threatening condition, with a high in-hospital mortality rate reaching 40%. In contrast, Type B IMH is less frequently associated with poor outcomes, showing an in-hospital mortality risk of <10% (2, 3, 5, 16).

Compared to AD, malperfusion syndrome is less common in patients with IMH, while periaortic hematoma and pericardial effusion are much more frequent (5). After initial detection and management of IMH, regular follow-up imaging studies with CT or MRI are performed to monitor resolution, stability, or progression.

**Figure 5:**
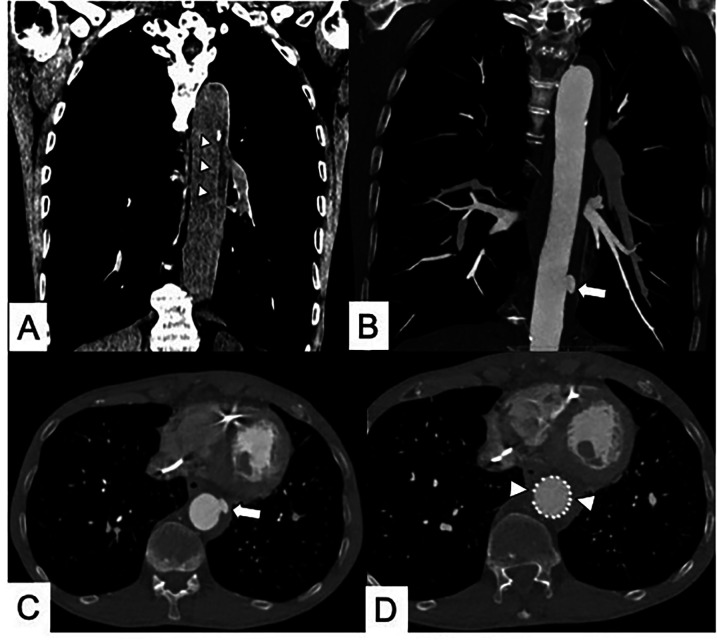
**(A)** Coronal multiplanar reformation CT in subacute Type B IMH (arrowheads). **(B,C)** One-month CT follow-up shows the appearance of an ulcer-like projection (ULP) treated **(D)** with thoracic endovascular aortic repair (TEVAR) (arrowheads).

IMH has an unpredictable evolution without treatment compared to AD. IMH may partially resolve or completely regress after the first 30 days or within 1 year and in some cases even 5 years from initial presentation. Resolution occurs in approximately 19% of Western patients and over 60% of Asian patients (18, 57–60). More frequently, conversion of IMH to AD may occur within the first 6–8 days (3); in 33%–40% of Type A IMH patients at hospital admission, the condition progresses to Type A AD while up to 18% are complicated by rupture (7), compared to Type B IMH which has a certainly more benign course even if not free from complications which can occur in approximately 2.5% of patients (Figure 5) (60).

Development of an aneurysm (saccular or fusiform) indicates a progressive weakening of all three layers of the aortic wall. In particular, structural weakness of the media determines the formation of the most common long-term complication of IMH, the fusiform aneurysm, typically during the subacute or chronic stages of the disease (60–63).

**Figure 6:**
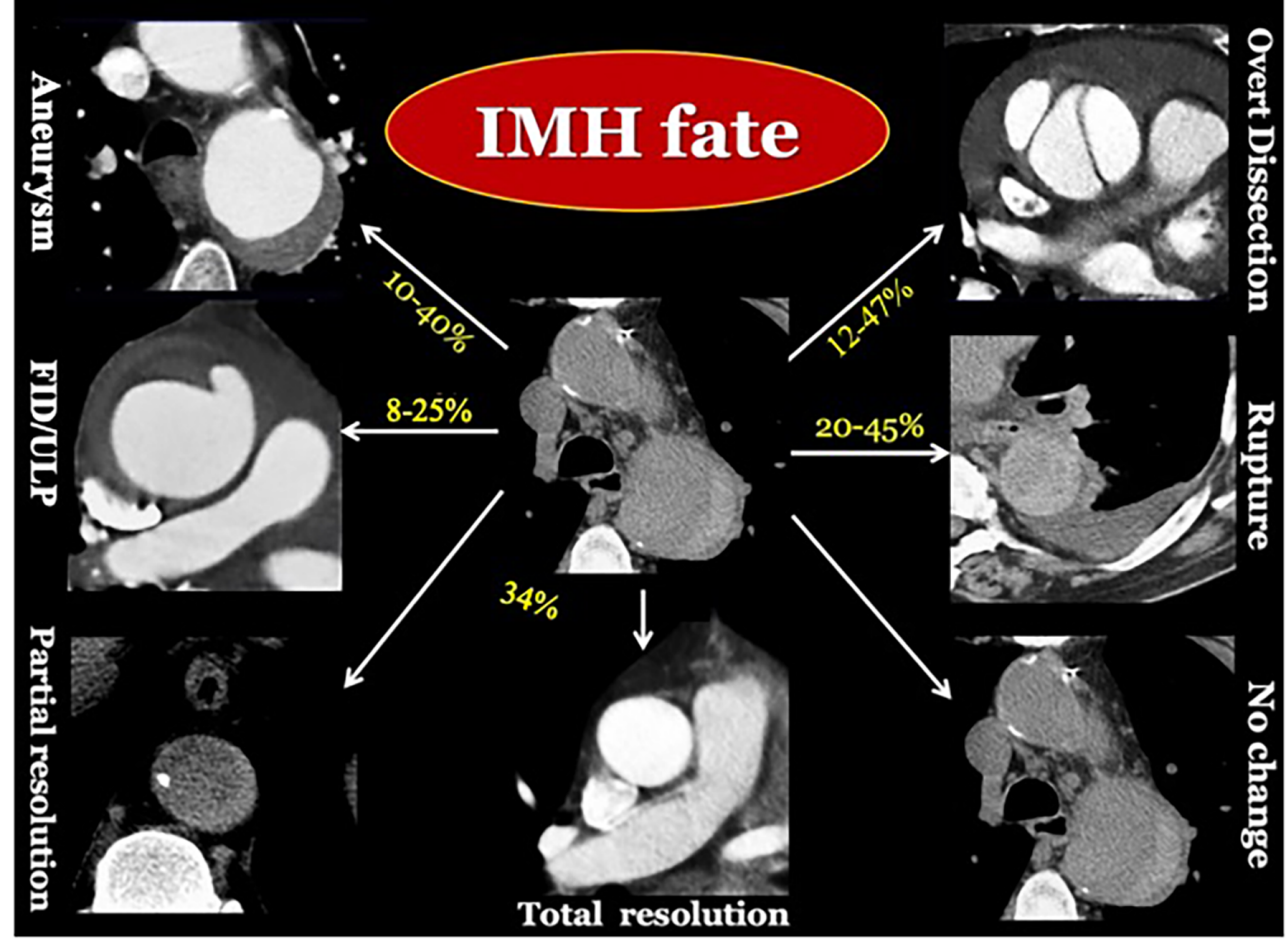
Longitudinal CT imaging of IMH evolving patterns. IMH is a vulnerable dynamic condition, with unpredictable late fate (16, 18, 57, 61).

These mutable natural progression patterns and remodeling processes in all phases of the disease highlight the increased vulnerability of the “aortic organ” and dynamic condition of IMH underscoring the critical need for close imaging surveillance to prevent progressive dilation or rupture, particularly within the first 30–60 days (Figure 6) (59–62, 64). Therefore, therapeutic decisions heavily rely on imaging, and for this reason, it is essential for the radiologist to recognize the CT high-risk features in time.

## High-risk IMH features

**Figure 7:**
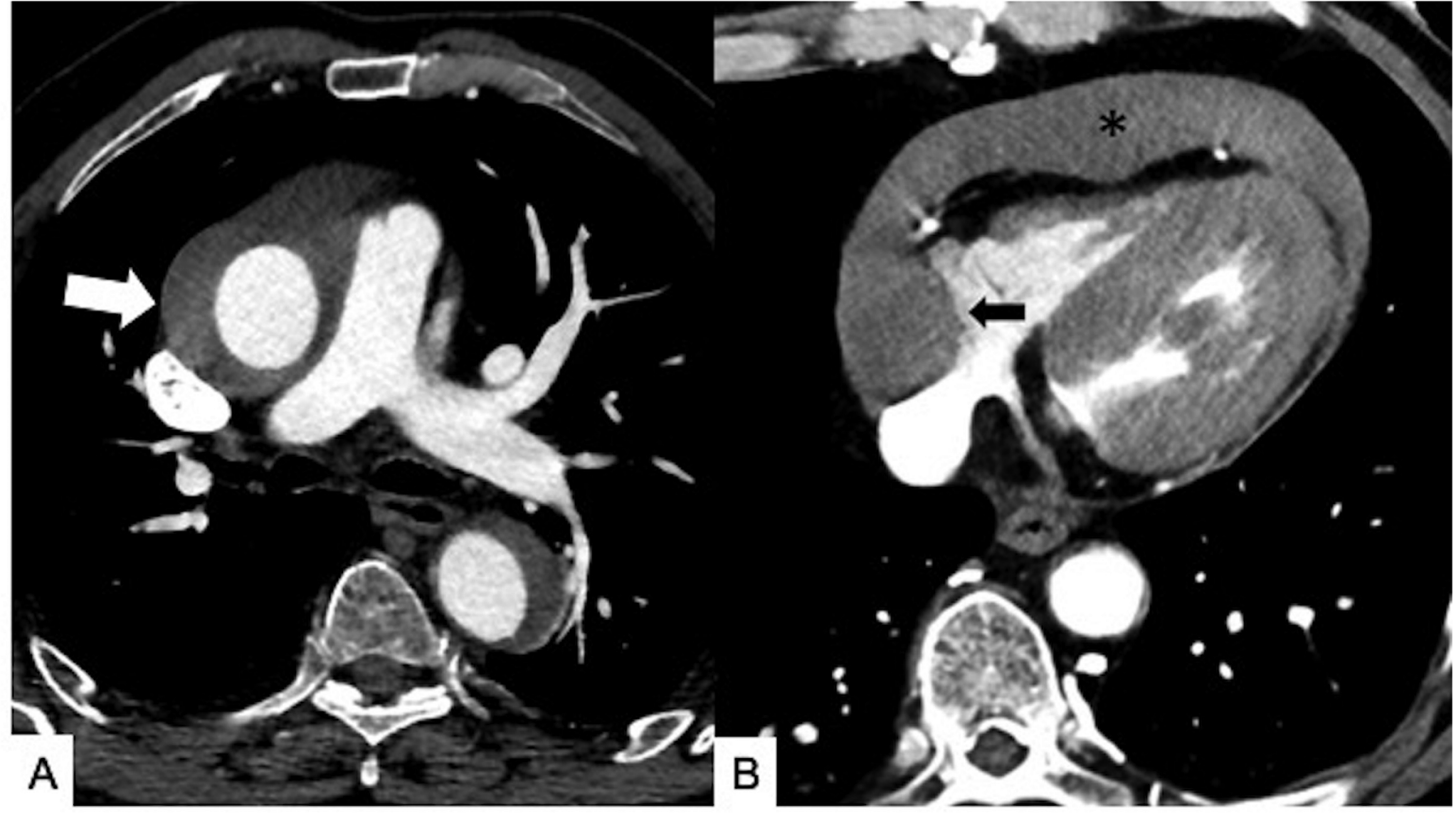
Maximal IMH thickness and pericardial effusion as poor prognostic factors in a 68-year-old male patient with acute back pain and dyspnea. **(A)** Axial contrast-enhanced CT scan shows a Stanford Type A IMH with hematoma thickness >10 mm (white arrow). **(B)** Axial contrast-enhanced CT image shows hemorrhagic pericardial effusion (asterisk) and mass effect (black arrow) on the right atrium suggesting impending cardiac tamponade.

**Figure 8:**
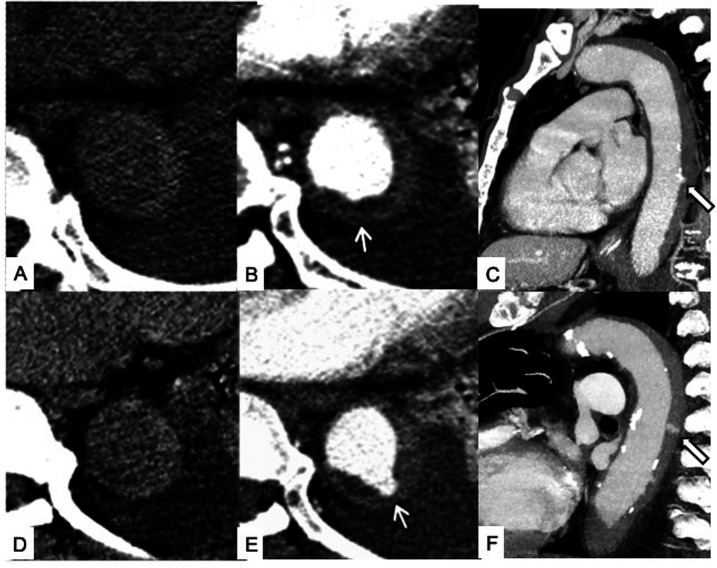
Temporal evolution of unstable Type B IMH. Initial unenhanced scan **(A)** and contrast-enhanced axial **(B)** and sagittal **(C)** CT images showing a Type B IMH with tiny intimal erosion of the descending aorta (white arrows). Four-day follow-up unenhanced scan **(D)** and contrast-enhanced axial **(E)** and sagittal **(F)** CT images at the same level showing enlargement of the intimal defect and enhancing ULP with neck >3 mm from the aortic lumen into the hematoma (white arrows), indicative of overt intimal tear and impending dissection.

**Figure 9:**
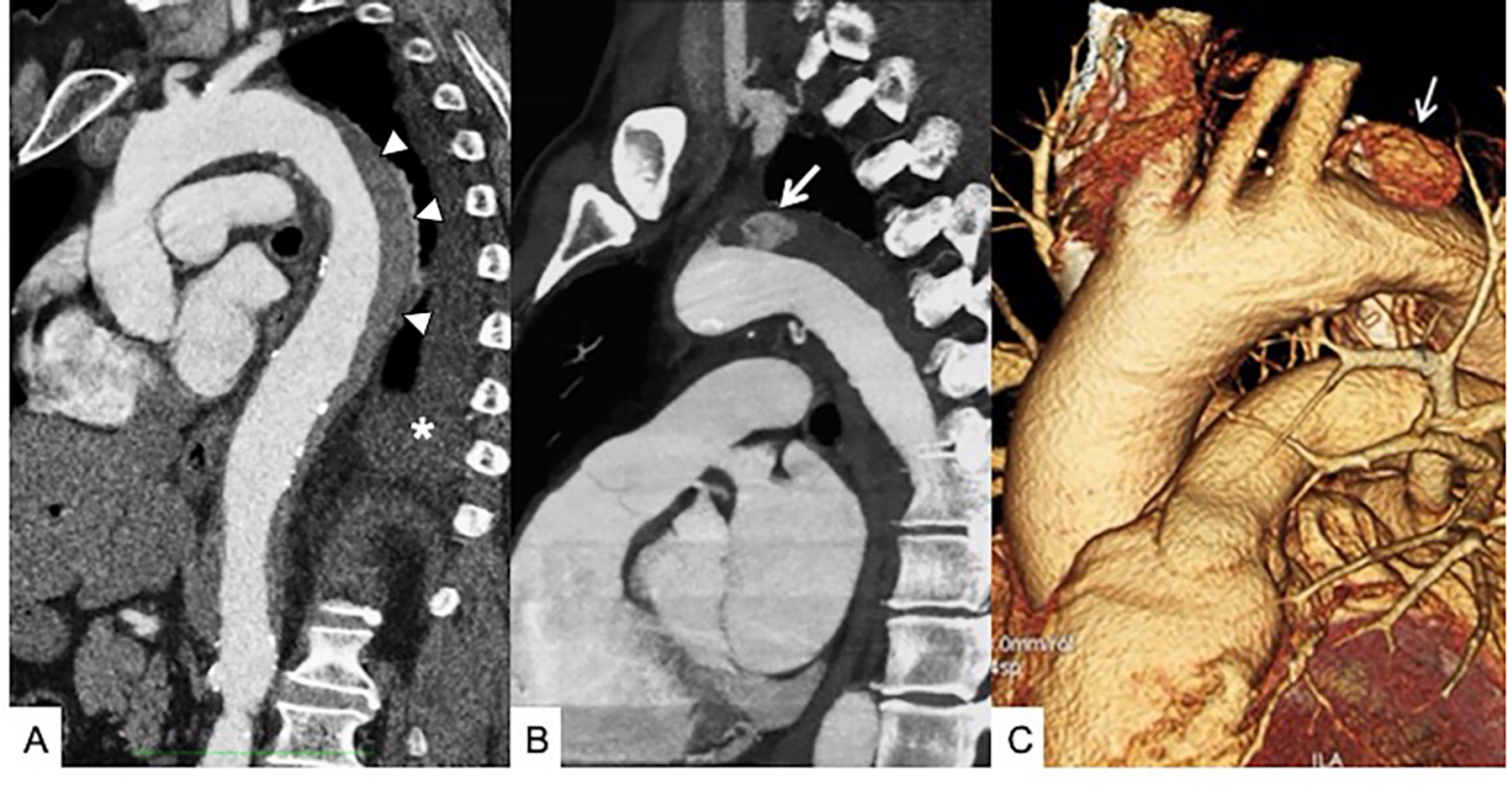
Unstable Type B IMH. **(A)** Sagittal contrast-enhanced baseline CT shows Type B IMH (arrowheads) in a 68-year-old male patient with hypertension peak and persistent thoracoabdominal pain; pleural effusion is present (asterisk). **(B)** Six-day follow-up contrast-enhanced CT sagittal multiplanar reformation shows the appearance of an ULP close to the aortic isthmus (arrow). **(C)** Sagittal oblique 3D volume rendering (VR) reconstruction confirms ULP (arrow). The patient undergoes TEVAR.

**Figure 10:**
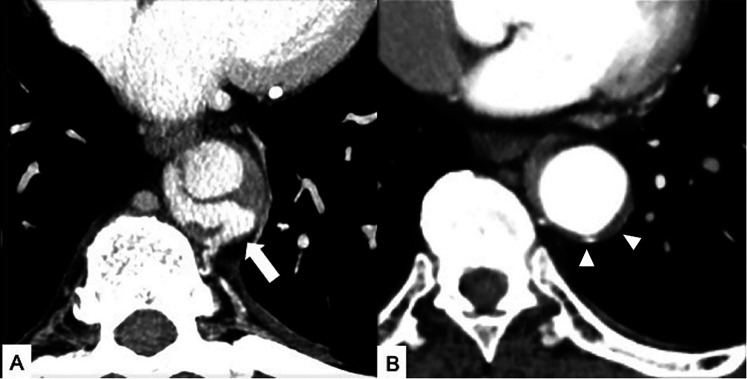
Intramural blood pool (IBP) in a 61-year-old patient with systemic hypertension and Type B thoracoabdominal IMH and acute back pain. **(A)** Axial CT maximum intensity projection (MIP) reconstruction image shows a T9 level intercostal artery pseudoaneurysm (arrow) in the contest of the Type B IMH with absent communication with the true aortic lumen. **(B)** Enhanced CT axial image obtained 3 months later shows a partial IBP and IMH regression (arrowheads).

The identification of some imaging prognostic features, as listed in Table 1 and below, can determine the cases of IMH at higher risk for complications or progression (61–65):

- The *differentiation according to Stanford classification* has prognostic implications. Approximately 30%–35% of cases are Stanford Type A IMH, which carries a higher risk of pleural or/and pericardial effusion, AD, aneurysmatic evolution, and death (53, 54, 62, 63).
- According to Laplace’s law and vascular wall stress, the *maximum aortic diameter* is an independent risk factor for complications, including rupture and death. Previous studies have stratified increased risk of complications based on the Stanford classification (48–55 mm for Type A IMH; 40–41 mm for Type B IMH). However, the recent European Association for Cardio-Thoracic Surgery/Society of Thoracic Surgeons Guidelines for Diagnosing and Treating Acute and Chronic Syndromes of the Aortic Organ, as suggested by Czerny et al., propose a cutoff diameter >45 mm, irrespective of the location (7, 65–67). Mazzolai et al., in 2024 ESC guidelines for the management of peripheral arterial and aortic diseases, consider a high-risk feature of a slightly larger maximum aortic diameter in IMH Type B (>47 mm) (18).
- The *maximum hematoma thickness* (>10 mm) decreases the chance of complete resorption, increasing the probability of progression (Figure 7) (7, 68–72).
- A *focal intimal disruption* characterized by a small pouch (<3 mm) filled with contrast material and projecting outside the opacified aortic lumen should always be identified on the baseline CT because it can rapidly develop into an *ulcer-like projection* (ULP) until a frank double-barrel dissection (Figure 8). A ULP is a new or already existing intimal disruption associated with a poorer prognosis (Figure 9) (72). Its incidence ranges from 20% to 60% (28, 73, 74) and can develop after the acute event from 2.4 to 17.8 months (67, 74). As a focal intimal disruption, ULP is a contrast material-filled pouch that projects outside the opacified aortic lumen and communicates with it through an orifice of the intimal layer >3 mm (28). ULP is differentiated from PAU because it is usually not visible on the initial CT scan but appears during follow-up imaging. Additionally, atherosclerotic disease is not associated with ULP. The detection of ULP already in the acute phase affects the prognosis making it poor especially if located in the ascending aorta or aortic arch and if the diameter exceeds 20 mm and depth reaches 10 mm. ULP often progresses to complications such as AD, saccular pseudoaneurysm, or rupture (67, 74). Indeed, the risk of adverse aortic events in the presence of ULP is reported to be 2.76 times higher compared to cases without ULP, and this risk increases to 3.84 times in patients with newly developed ULP (71, 72, 75). The simultaneous overlapping of different types of AAS (aortic dissection, IMH, ULP, and PAU) in an individual is classified as a mixed-type aortic lesion, which poses additional challenges in interpretation.
- *Intramural blood pool* (IBP) or aortic branch artery pseudoaneurysms are focal areas of contrast pooling within the hematoma that communicates with the lumen of the aorta through the ostia of intercostal or lumbar arteries that have been disrupted by the hematoma. These are typically found exclusively along the non-pleural circumference of the descending aorta, at the origin of aortic side branches such as bronchial, intercostal, intercostobronchial, pericardial, or lumbar arteries. Communication with the aortic lumen is usually very small (<2 mm) or imperceptible. IBP are generally considered benign features even if their prognostic significance is uncertain and currently the studies performed are limited (Figure 10). IBP occurs due to damage from IMH extending across the origin of the aortic branch artery, resulting in partial or complete tear. IMH with multiple IBP at various levels is reported as the “Chinese ring sword sign” on CT coronal reconstructions of the descending aorta (76). IBPs larger than 10 mm or that are enlarging may require endovascular treatment (77).

**Table 1:** Imaging and clinical features indicative of a high risk of progression.

› Ascending aorta involved (Type A IMH)
› Aortic diameter >5 cm (a greater stress on the dilated aortic wall implies a greater risk of rupture)
› Hematoma thickness (HT) >10 mm
› New or already existing ULP
› Luminal compression ratio (minimum/maximum transverse luminal diameters at the site of the maximal HT)
› Associated PAU (penetrating atherosclerotic ulcer), diameter >20 mm and depth >10 mm
› Temporal aortic enlargement on serial imaging (rapid aortic diameter growth during hospital stay)
› Periaortic, pleural, or pericardial effusions, particularly if large or temporally progressive
› Persistent pain, hemodynamic instability, or both

Additional high-risk features for progression and mortality include age over 70 years, pleural or pericardial effusion based on Hounsfield units (values >40–60 Hounsfeld units suggest acute/subacute hemorrhage) (78), and periaortic hematoma (7, 67). Furthermore, some previous studies have indicated that a luminal compression ratio (the minimum to maximum transverse luminal diameters at the site of maximal hematoma thickness determined by three-dimensional double-oblique MPR reconstruction) that is <0.75 is also predictive of IMH progression in aortic dissection (79, 80).

## Treatment and imaging follow-up

The treatment of IMH has always been controversial. Upon diagnosis, all patients receive immediate medical treatment focused on minimizing stress on the aortic wall, mainly using β-blockers to manage heart rate, ventricular contractility, and systemic blood pressure, as well as pain control. Subsequent treatment strategies depend on the affected aortic segment as determined by the Stanford classification. Over the years, Type A IMH has always been considered a surgical emergency, based on evidence showing a lower mortality rate with early surgical intervention compared to a 40%–80% mortality rate in patients who did not undergo surgery. Although some reports from Asian cohorts have considered initial non-surgical treatment for this condition, reserving surgical treatment only to patients who experience complications (60, 65, 66, 73), and some surgeons advocate for urgent rather than emergent surgery—assuming that delayed treatment can reduce inflammation making the aortic tissue more manageable for repair—this strategy appears to offer no clinical advantage (81).

The recent Guidelines for Diagnosing and Treating Acute and Chronic Syndromes of the Aortic Organ recommend surgical intervention for all patients exhibiting high-risk type A IMH features, as emergency surgical repair has shown superior outcomes compared to medical therapy alone (7).

For patients lacking high-risk features or who are elderly or have significant comorbidities, a “wait-and-watch strategy” involving non-operative management with pain control and frequent imaging follow-up may be considered a viable option (7, 82, 83).

On the contrary, the initial choice for Type B IMH is non-operative management, reserving thoracic endovascular aortic repair (TEVAR) to the complicated acute Type B IMH or in cases with CT high-risk features (e.g., enlargement of aortic diameter, appearance of ULP, hematoma progression to frank dissection, or visceral ischemia) and would include coverage of the entire diseased segment. Surveillance imaging in patients with non-surgically treated Type B IMH or surgically treated IMH is similar to that in patients with classic Type B AD (13).

In IMH, both progression and regression are possible over the first 30 days to 1 year or as long as 5 years after the onset (59), so a CT or MR evaluation at discharge and at 1, 3, 6, and 12 months after the acute event is necessary and, if not resolved, annually for the next 5 years in which it is possible to have a further aortic event (5, 16, 64).

## Main differential diagnosis

IMH is easily distinguished from *classical AD* by the absence of an intimomedial tear or flap in the acute phase and the lack of direct flow communication. Diagnosis can often be challenging if the AD false lumen is completely thrombosed, and, in these cases, the direct identification of the entry tear may be possible only after surgery or autopsy. Furthermore, while IMH maintains a constant circumferential relationship with the aortic wall, the dissection describes a spiral longitudinally (23). In *incomplete AD* (Svensson Class III of AAS) in which in the presence of an intimomedial rupture the separation of the medial layers does not occur (2, 84), typical features are an eccentric bulge near the tear and concomitant aortic dilation (1, 2, 13, 17, 84).

The presence of *atherosclerotic changes* or *aneurysmal dilation with mural thrombi* also makes the differential diagnosis particularly challenging. The presence of non-hyperattenuating wall thickening on non-enhanced CT, an irregular internal contour in the arterial phase, and irregular, thick calcifications should guide the radiologist toward diagnosing atheromatous changes rather than IMH, which typically presents with smooth inner margins and thin, curvilinear intimal calcification displacement (1, 7, 17, 21, 33, 34).

**Figure 11:**
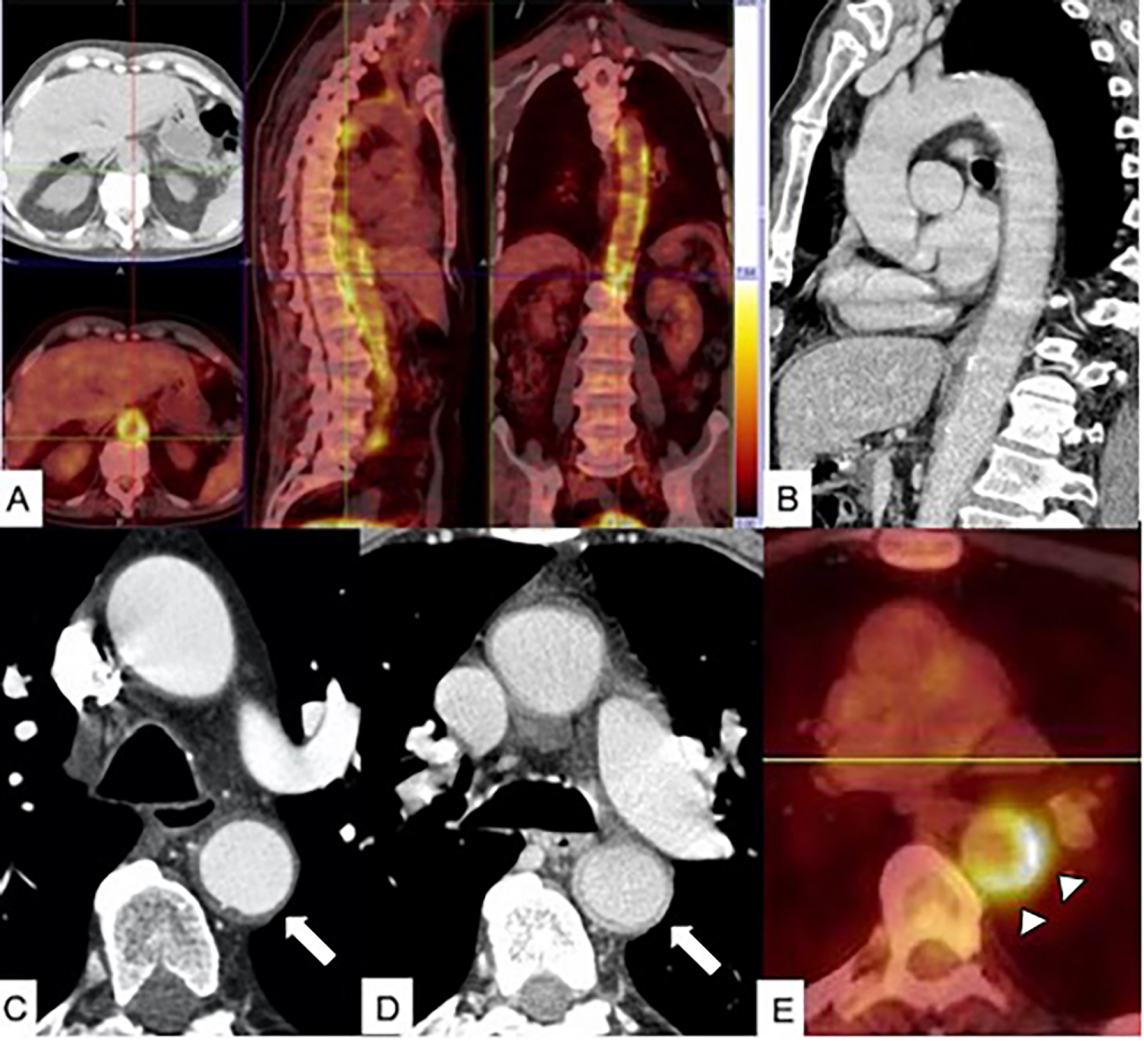
PET/CT in aortitis. **(A)** Axial PET/CT fusion image in a 68-year-old male patient with diffuse aortitis showing an [18F]-fluorodeoxyglucose (FDG) uptake in the thoracoabdominal aortic wall (arrow). **(B)** Sagittal multiplanar reconstruction CT image acquired in the portal phase shows thickened aortic wall enhancement. In a different 70-year-old female patient with giant cell arteritis, arterial-phase CT scan **(C)** shows thickening of the descending aortic wall mimicking an IMH (arrow), and venous phase CT image **(D)** shows enhancement of thickened aortic wall (arrow). **(E)** Axial PET/CT fusion image in the same patient shows FDG uptake of the descending aorta wall (arrowheads).

*Infectious and non-infectious aortitis* (e.g., giant cell arteritis or Takayasu’s arteritis) can present with uniform and hyperattenuating wall thickening on non-contrast CT scans, posing a challenge in differentiating from IMH. Aortitis often manifests with gradual narrowing of the distal aortic segment, creating a characteristic “rat-tail” sign. Additionally, the aortic wall typically enhances in the portal venous phase following contrast medium administration and may exhibit features such as transmural calcification or a double-ring enhancement pattern. This pattern consists of a poorly enhanced inner ring (representing the swollen intima) and an enhanced outer ring (indicating active inflammation in the medial and adventitial layers) (85). In case of aortitis and in non-emergency settings, diffuse [18F]-fluorodeoxyglucose (FDG) uptake in areas of active inflammation in the aortic wall will be present on FDG positron emission tomography (PET) allowing not only to distinguish AAS from inflammatory and infectious aortitis but also to evaluate the effectiveness of the therapy (Figure 11) (86). Furthermore, in IMH, the aortic lumen is slightly narrowed, and a definite transition between the involved and normal aorta is clearly appreciable, a feature not typically seen in aortitis and thrombus (67, 87). Lastly, the absence of acute chest pain should help distinguish inflammation from intramural thrombus or IMH which typically presents with marked painful symptoms.

*Acute contained rupture* of an abdominal or thoracic aortic aneurysm may mimic acute IMH, as both conditions exhibit a high-attenuation crescent sign. However, while IMH begins in the aortic media, in acute contained rupture, high attenuation and CM infiltration begin from the aneurysm lumen through the intramural thrombus and may later penetrate the wall. In the case of *chronic contained rupture*, CT features can include discontinuity of the rim of calcification in the aneurysm wall, well-defined soft tissue density adjacent to the aorta, psoas muscle involvement, displaced abdominal structures, and no appearance of contrast material in the hematoma (88). Furthermore, the association with the erosion of the vertebral bodies is frequent as recently reported by Parillo et al. (89). In both acute and chronic aortic contained rupture, the “draped aorta sign” can be present in which the aortic posterior wall is not identifiable from adjacent structures or molds the contour of adjacent vertebral bodies; identifying this sign is also useful for differentiating aortic contained rupture from extra aortic pathologies, such as retroperitoneal tumors and osteomyelitis (90, 91).

*Aortic sarcoma*, a very rare disease with intimal or mural origin, presents with CT features similar to those of IMH. In particular, it can present crescent-shaped thickening of the thoracic aorta wall with the same attenuation of the aortic lumen and displacement of intimal calcification; however, in the presence of an irregular polypoid mass within the aortic lumen with lobulated contour, extravascular tissue growth, and neoplastic enhancement, sarcoma is an eventuality to keep in mind. Embolization of distant organs is common. Diagnosis is almost always by postoperative histopathology and immunohistochemical markers.

The main differential diagnoses of IMH are listed in Table 2.

**Table 2:** CT key features in the differential diagnosis of IMH.

Aortic disease	CT key features
Aortic dissection	Intimal tear, intimomedial flap, false lumen
AD with FL totally thrombosed	Possible FL with a spiral course, often no differential diagnosis by imaging
Incomplete dissection	Eccentric bulge near the tear, focal aortic dilation
Atherosclerotic changes	No hyperattenuating thrombus, no displaced intimal calcium, irregular inner margin
Aortitis	Concentric double-ring enhancement (portal phase), PET positive
Contained aneurysm rupture	Acute (high-attenuation and acute bleeding through the mural thrombus); chronic (draped aorta sign, vertebral bodies erosion)
Aortic sarcoma	Lobulated contour may extend beyond the wall, neoplastic enhancement, possible embolization

AD, aortic dissection; FL, false lumen.

## Conclusion

Today, IMH remains a frequently challenging and potentially life-threatening diagnosis. Therefore, the aim of this review is not only to describe its CT features but also to emphasize the concept that the aortic organ should be considered as an independent “functional unit” where every single component of its structure (vasa vasorum/adventitia, media, or intima) can be affected by different pathologies, often interconnected, overlapping, or even existing simultaneously, but in most cases benefiting from similar management approaches. Despite the ongoing controversy surrounding IMH pathogenesis, an emergency radiologist, especially one who works in small community hospitals without highly specialized cardiothoracic departments and who suspects AAS, must promptly identify high-risk CT features of IMH and centralize patients in a reference “aorta center” to ensure that they receive the most suitable treatment. In acute settings, non-invasive CT scans have contributed to a better understanding of AAS as well as IMH and its evolving nature that therefore requires serial control CT scans with dose-saving techniques both during hospitalization and after discharge. Decision-making should be individualized depending on IMH location, appearance, dimensions, and associated pathology; moreover, case discussion by a multidisciplinary team is mandatory to reduce early mortality and avoid reoperations improving long-term outcomes.
